# Determinants of early childhood morbidity and proper treatment responses in Vietnam: results from the Multiple Indicator Cluster Surveys, 2000–2011

**DOI:** 10.3402/gha.v9.29304

**Published:** 2016-02-29

**Authors:** Hwa-Young Lee, Nguyen Van Huy, Sugy Choi

**Affiliations:** 1 JW Lee Center for Global Medicine, Seoul National University, College of Medicine, Seoul, Korea; 2 Department of Health Management, Institute for Preventive Medicine and Public Health, Hanoi Medical University, Hanoi, Vietnam

**Keywords:** cough, diarrhea, MICS, oral hydration therapy, prevalence, under-five children, Vietnam

## Abstract

**Background:**

Despite significant achievements in health indicators during previous decades, Vietnam lags behind other developing countries in reducing common early childhood illnesses, such as diarrhea and respiratory infections. To date, there has been little research into factors that contribute to the prevalence and treatment of childhood morbidity in Vietnam.

**Objective:**

This study examines the determinants of diarrhea and ‘illness with a cough’ and treatments for each of the conditions among young children in Vietnam, and describes trends over time.

**Design:**

Data from the Vietnam Multiple Indicator Cluster Surveys in 2000, 2006, and 2011 were used. Multivariable logistic regressions were undertaken to investigate factors associated with these childhood illnesses and proper treatment patterns.

**Results:**

Between 2000 and 2011, the prevalence of diarrhea among children under the age of five declined from 11 to 7%, while having illness with a cough increased to 40% in 2011 after falling from 69 to 28% between 2000 and 2006. During the same period, the prevalence of oral rehydration therapy (ORT) for treating diarrhea increased from 13 to 46%, whereas the rate of seeking formal treatment for illnesses with a cough fell from 24 to 7%. Multivariable models indicated that children who were older than 2 years (odds ration [OR]: 0.44, 95% confidence interval [CI]: 0.37–0.53, *p<*0.001), male (OR: 1.21, 95% CI: 0.64–2.37, *p<*0.05), living in rural areas (OR: 1.28, 95% CI: 1.00–1.64, *p<*0.05), or of Kinh ethnicity (OR: 0.70, 95% CI: 0.56–0.87, *p<*0.01) were more likely to suffer from diarrhea. Ethnic differences and higher household wealth were factors significantly associated with having illness with a cough. In particular, the effect of level of wealth on illness with a cough varied in each wave. Mothers with higher levels of education had higher odds of seeking ORT compared with mothers with the lowest level of education. Seeking formal treatment for children who have illness with a cough was associated with being in a household in the richest wealth quintile (OR: 0.56, 95% CI: 0.34–0.91, *p<*0.05).

**Conclusions:**

This study demonstrates the importance of identifying different risk factors for these two illnesses and also factors associated with healthcare-seeking behaviors in order to reduce the burden of childhood morbidity in Vietnam. Policies aimed at tackling childhood morbidities should include comprehensive strategies that impact on socioeconomic and environmental factors.

## Introduction

Epidemiological evidence suggests that diarrhea and respiratory infections are main causes of morbidity and mortality in children under the age of five, accounting for more than two-fifths of all deaths worldwide (1). Diarrheal disease is the third leading cause of infant and child mortality in developing countries and represents 11% of all deaths in children under the age of five (2). One major reason for the poor outcomes of diarrhea episodes is the mismanagement of cases during acute situations. The use of rehydration therapy for watery diarrhea in children is directly related to survival (3). When a child is suffering from moderate dehydration, oral rehydration solution (ORS) may be enough while intravenous infusion of rehydration solution is needed in more severe cases (4).

Oral rehydration therapy (ORT) has significantly contributed to the reduction of childhood deaths from diarrhea (5) being operationally simple, effective, and inexpensive (6). However, despite its salient benefits, the use of ORT for diarrhea in developing countries has been neglected for various reasons. Many misconceive the seriousness of diarrhea in children and overlook the benefits of ORT treatment believing that every child normally experiences diarrhea and that they will recover without treatment (7). Some parents do not use ORT for reasons of affordability and accessibility (7). These barriers to the use of ORT impact differently on population subgroups making it difficult to correctly identify the most vulnerable.

Manna et al.'s study of the determinants of healthcare-seeking behavior for diarrheal illness among children under the age of five in India found that the formal education of primary caregivers was associated with care seeking outside the home (8). Another study by Ali et al. (9) observed that misperception of diarrhea in rural Bangladesh was the most common barrier to the use of ORT among severe cases and that difficulties in administering ORS were also common. In one of the few studies of ORT implementation in the Vietnamese context, Kaljee et al. investigated the preferences for healthcare providers regarding diarrhea treatment in Nha Trang. Using both qualitative and quantitative analysis, they showed that more than half of the respondents self-treated with medication purchased from a pharmacy or medications stored at home during the initial stages of diarrhea. Only less than a quarter utilized public healthcare providers during the initial stage of illness (10).

Childhood pneumonia is another major cause of childhood mortality. Each year, more than two million children die of acute respiratory infections, mostly pneumonia (11). Rapid recognition and treatment with effective medicine is critical because fatality when untreated is extremely high (12). In addition, the infection can be mistaken for malaria because the symptoms are similar (13). For these reasons, it is very important to obtain accurate diagnosis and deliver treatment quickly by an appropriate healthcare provider once the initial symptoms are apparent.

There are few studies on determinants of the prevalence or treatment of respiratory illnesses with cough symptoms in children. Hatt et al. explored the interaction between parental education and economic status on the prevalence of respiratory illness (14). They found that maternal education was more protective of the disease for children in wealthy families than for children in poor families, while father's education was also protective but operated independently of economic status. Basu et al. also emphasized the importance of maternal education. They showed that lack of maternal education is a significant correlate of cough episodes in children and that even primary education completion is protective of child health (15). However, to the best of our knowledge, apart from one qualitative study (10), little if any research has been published on healthcare-seeking behaviors for treating respiratory illnesses with cough symptoms in children in Vietnam.

Because of the seriousness of diarrhea and respiratory infections in children in developing countries such as Vietnam, research is needed to investigate the determinants of occurrence of those illnesses and also barriers to seeking treatment for these conditions. Given the major social and economic transitions that Vietnam has experienced in recent years, it is important to consider how patterns of illness and treatment are changing over time. This epidemiological study of children in Vietnam has two main objectives. The first is to identify the determinants of diarrhea and ‘illness with a cough’ and assess change in the determinants over time. The second objective is to identify factors that determine whether treatment is sought for these two conditions and assess change in these factors over time.

## Methods

### Data source

Data were derived from the three rounds of Multiple Indicator Cluster Survey (MICS) in Vietnam, performed in 2000 (wave 2), 2006 (wave 3), and 2011 (wave 4), respectively. The MICS was initially designed by UNICEF and then conducted by the General Statistics Office in collaboration with the Ministry of Health (MOH) and the Ministry of Labor, Invalids and Social Affairs (MOLISA). The surveys are intended to provide internationally comparable and statistically rigorous nationally representative data to fill gaps in evidence on issues related to the health status, development, and living standards of Vietnamese women and children.

The sample for MICS in Vietnam was based on probability, using the urban and rural areas within each of six regions as the main sampling strata. Sampling was undertaken in two stages. Within each stratum, a pre-determined number of enumeration areas were chosen using the probability proportional to the size. After household lists were updated within the selected enumeration areas, systematic samples of 20 household were drawn in each sample enumeration.

The MICS is a repeated cross-sectional survey, which targets different respondents each year. Three waves of data collection (2nd, 3rd, and 4th wave) in 2000, 2006, and 2011 are currently available. The MICS datasets and results have been published on the UNICEF website (mics.unicef.org). There are three separate datasets in each wave, which are for households, women aged 15–29 years, and children under the age of five. Data used in this analysis were drawn from the dataset for children. More detailed information on MICS can be found elsewhere (16–18).

### Dependent variables

There are four binary dependent variables, two measuring recent illnesses and two measuring treatment for those who reported the illnesses. The illness variables are diarrhea and illness with a cough. They were derived from answers to questions about whether a child aged 5 years or less had experienced either diarrhea or an illness with a cough during the 2 weeks prior to the interview. Response options were yes versus no.

The treatment measures refer to ORT for recent diarrhea and formal treatment for recent illness with a cough. The ORT variable was derived from answers to a question, which asked mothers about treatment given to children during a recent episode of diarrhea. This question had five possible responses, which were used to derive a binary variable: fluid from ORS packet or pre-packaged ORS fluid versus other kinds of water. The binary treatment variable for recent illness with a cough was derived from responses to a question, which asked about the treatment mothers sought. This variable was coded: seeking formal healthcare such as public or private hospital, health center, or doctor versus seeking relative/friend, shop, traditional healer, or none.

### Independent variables

Demographic and socioeconomic factors available in the MICS dataset, which were known, from previous studies, to have association with the dependent variables, were chosen as the independent variables. Child's age, in years, was transformed into a binary variable using the mean age of the study population as the cutoff point. Breastfeeding was dichotomized as yes (ever breastfed) versus no (never breastfed). The mother's education variable comprised five categories: ‘less than primary’, for respondents who had never been to school or did not finish grade 5; ‘lower secondary’, for respondents who attained their highest level of education between grades 6 and 9; ‘upper secondary’, for respondents who attained their highest level of education between grades 10 and 12; and ‘tertiary’, for respondents who finished professional school, college, or university and above.

Ethnicity was classified into two groups: Kinh, which is the ethnic majority in Vietnam, accounting for about 86% of the population, and non-Kinh (18). A household wealth index was used as a proxy variable for economic status. This was constructed based on the information on household's ownership of consumer goods and amenities related to dwelling characteristics, water, and sanitation. Household wealth scores were derived by principal component analysis, and the scores were divided into quintiles from the poorest to the wealthiest. A time variable (wave) was included as a proxy for survey year.

### Statistical analysis

Descriptive statistics were conducted to describe patterns of illness and treatment by the independent variables in each separate survey wave (2000, 2006, and 2011). Using a combined dataset covering all three survey waves, separate sets of multivariable logistic regressions were performed for each of the four dependent variables. Odds ratios (OR) and 95% confidence intervals (CIs) are reported. Initially, four separate models with only main effects were examined. Additional analyses were performed including interaction terms (time multiplied by each predictor variable) to assess change in associations between independent and dependent variables across the survey waves. Each interaction term was tested separately, and only those that were significant at *p*≤0.05 were included in the final model. All analyses were performed using STATA 12.0 software.

### Ethical consideration

This study is based on secondary data from the MICS with all identifying information that has been removed. The survey obtained informed consent from the mothers before conducting survey questionnaires. All information in the original dataset was collected confidentially (16–18).

## Results

### Derivation of study sample

**Fig. 1:**
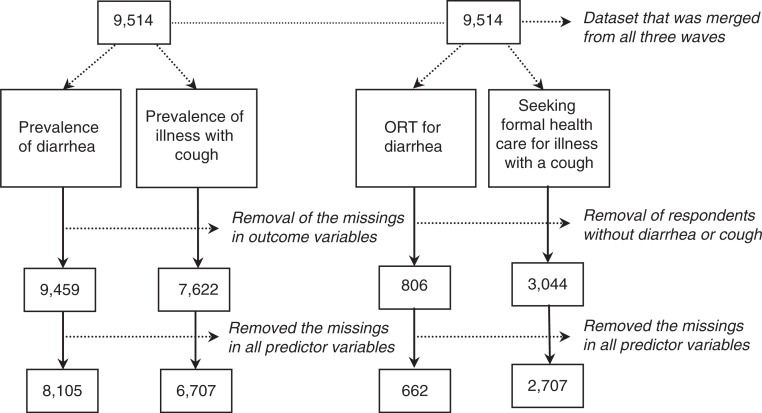
Derivation of study samples for logistic regression analysis using combined MICS data, 2000, 2006, and 2011.

Figure 1 shows the derivation of the four study samples used in the regressions. The initial study population (*N*=9,514) was combined from three waves of MICS including complete data on the relevant dependent and independent variables.

### Patterns of illness, treatment by survey years

Tables 1 and 2 show the prevalence of four dependent variables by demographic and socioeconomic characteristics. Diarrhea was much less common than illness with a cough (11% vs. 69% in 2000, 8% vs. 28% in 2006, and 7% vs. 40% in 2011). Prevalence of diarrhea decreased continuously from 11% in 2000 to 7% in 2011, whereas the prevalence of an illness with a cough rebounded to 40% in 2011 after declining from 69% in 2000 to 28% in 2006. There was a consistent pattern in the prevalence of diarrhea across the three waves. For example, children who were older, were male, had ever been breastfed, had mothers with higher levels of education lived in urban areas, or were of Kinh ethnicity had lower prevalence of diarrhea than their counterparts in all 3 years (2000, 2006, and 2011). However, illness with a cough was less consistent across the survey waves (Table 1).

**Table 1:** Distribution of diarrhea and illness with cough in children under-five in Vietnam by demographic and socioeconomic characteristics, 2000, 2006, and 2011

		Diarrhea						Illness with cough					
			
		2001		2006		2011		2001		2006		2011	
							
Variables	Category	*N*	%	*N*	%	*N*	%	*N*	%	*N*	%	*N*	%
Average		2,471	11	2,251	8	3,383	7	1,072	69	2,251	28	3,384	40
Child's age	<2 years	1,371	13	1,325	10	2,058	9	627	70	1,325	29	2,059	39
	≥2 years	1,100	7	926	5	1,325	4	445	68	926	26	1,325	40
Child's gender	Female	1,209	9	1,105	7	1,652	7	531	71	1,105	27	1,653	38
	Male	1,262	12	1,146	9	1,731	7	541	67	1,146	28	1,731	41
Breastfeeding	No	46	9	38	3	76	7	22	68	38	24	76	45
	Yes	2,425	11	2,213	8	3,307	7	1,050	69	2,213	28	3,308	39
Mother's education	<Primary	818	13	380	9	670	8	365	70	380	22	671	42
	Lower secondary	1,239	11	347	11	1,379	7	556	70	347	26	1,379	40
	Upper secondary	351	7	847	8	660	7	125	64	847	31	660	37
	Tertiary	63	3	677	6	674	6	26	62	677	28	674	38
Area	Urban	495	7	332	4	1,373	6	199	62	332	27	1,373	39
	Rural	1,976	11	1,919	9	2,010	8	873	70	1,919	28	2,011	40
Ethnicity	Non-Kinh	400	14	746	12	481	11	154	65	746	24	481	39
	Kinh	2,071	10	1,505	6	2,902	6	918	70	1,505	30	2,903	40
Wealth quintiles	Poorest	642	14	757	10	667	9	300	67	757	23	667	44
	Second	606	13	468	5	571	7	278	73	468	32	572	42
	Middle	444	7	431	7	639	7	208	67	431	29	639	38
	Fourth	407	9	365	9	733	6	150	70	365	33	733	39
	Richest	372	12	230	3	773	6	136	65	230	23	773	35

**Table 2:** Distribution of ORT and formal treatment for cough in children under-five in Vietnam by demographic and socioeconomic characteristics, 2000, 2006, and 2011

		ORT						Formal treatment for illness with cough					
			
		2001		2006		2011		2001		2006		2011	
							
Variables	Category	*N*	%	*N*	%	*N*	%	*N*	%	*N*	%	*N*	%
Average		263	13	174	22	225	46	742	24	625	24	1,340	7
Child's age	<2 years	185	15	131	24	179	46	438	24	383	25	804	7
	≥2 years	78	9	43	16	46	46	304	24	242	21	536	7
Child's gender	Female	110	12	78	18	108	38	366	24	300	21	633	6
	Male	153	14	96	26	117	53	376	24	325	26	707	8
Mother's education	<Primary	104	9	33	6	51	25	254	23	82	27	285	7
	Lower secondary	133	17	37	27	94	51	389	26	91	23	555	8
	Upper secondary	23	13	65	25	42	52	80	19	263	19	244	6
	Tertiary	3	0	39	28	38	53	19	16	189	29	256	6
Area	Urban	37	19	13	31	74	47	124	19	89	17	539	5
	Rural	226	12	161	22	151	45	618	25	536	25	801	8
Ethnicity	Non-Kinh	56	14	84	15	51	43	102	25	179	27	188	5
	Kinh	207	13	90	29	174	47	640	24	446	22	1,152	7
Wealth quintiles	Poorest	93	9	76	18	56	32	204	29	175	26	294	8
	Second	63	11	35	20	41	41	204	24	149	23	243	7
	Middle	45	20	29	28	42	60	140	21	125	29	246	7
	Fourth	36	17	26	27	44	52	105	22	122	20	283	9
	Richest	26	15	8	38	42	48	89	19	54	13	274	4

The prevalence of ORT for diarrhea increased from 13% in 2000 to 22% in 2006, and 46% in 2011, while the proportion seeking formal treatment for illness with a cough decreased during the same period. In particular, there was a clear decline between 2006 and 2011. Prevalence patterns for both ORT utilization and formal treatment for illness with a cough varied across the three time points (Table 2).

### Factors associated with illness and treatment

Table 3 shows the results of the multivariable logistic regressions for each of the four dependent variables conducted on the dataset comprising multiple waves of the MICS (2000, 2006, and 2011). The odds of diarrhea and illness with a cough were lower in waves 3 and 4 compared to wave 2, holding all other variables constant. Children aged two or above (OR=0.44, CI =0.37–0.53) and of Kinh ethnicity (OR=0.70, CI=0.56–0.87) demonstrated lower odds of suffering from diarrhea compared with their counterparts. Male children and those living in rural areas had significantly higher odds of having recent diarrhea, compared to children who were female and living in urban areas.

**Table 3:** Multivariable regression results for the determinants of illnesses and proper treatments in children under-five in Vietnam, 2000, 2006, and 2011

		Diarrhea	Illness with cough	ORT	Seeking formal healthcare for illness with cough
Variables	Categories	OR(CI)	OR(CI)	OR(CI)	OR(CI)
Wave†	Wave 2	Ref.	Ref.	Ref.	Ref.
	Wave 3	**0.70 (0.55–0.88)	***0.21 (0.17–0.26)	1.78 (0.97–3.26)	0.98 (0.72–1.34)
	Wave 4	***0.67 (0.55–0.81)	***0.43 (0.33–0.57)	***5.26 (3.25–8.52)	***0.26 (0.19–0.34)
Child's age	<2 years	Ref.	Ref.	Ref.	Ref.
	≥2 years	***0.44 (0.37–0.53)	0.97 (0.87–1.08)	0.84 (0.52–1.35)	0.95 (0.76–1.18)
Child's gender	Female	Ref.	Ref.		Ref.
	Male	*1.21 (0.64–2.37)	1.05 (0.95–1.16)	*1.61 (1.09–2.38)	1.12 (0.90–1.39)
Mother's education	<Primary	Ref.	Ref.	Ref.	Ref.
	Lower secondary	1.02 (0.83–1.26)	0.99 (0.86–1.15)	***2.71 (1.56–4.71)	1.17 (0.88–1.55)
	Upper secondary	0.90 (0.69–1.17)	1.02 (0.86–1.21)	*2.26 (1.17–4.39)	0.85 (0.58–1.23)
	Tertiary	0.77 (0.56–1.06)	1.02 (0.85–1.24)	*2.32 (1.09–4.96)	1.33 (0.89–1.90)
Area	Urban	Ref.	Ref.	Ref.	Ref.
	Rural	*1.28 (1.00–1.64)	0.97 (0.85–1.12)	0.87 (0.50–1.51)	1.32 (0.95–1.83)
Ethnicity	Non-Kinh	Ref.	Ref.	Ref.	Ref.
	Kinh	**0.70 (0.56–0.87)	**1.29 (1.10–1.51)	0.71 (0.42–1.20)	1.07 (0.78–1.46)
Wealth quintiles	Poorest	Ref.	Ref.	Ref.	Ref.
	Second	0.87 (0.69–1.11)	*1.91 (1.17–3.11)	1.29 (0.72–2.32)	0.81 (0.60–1.11)
	Middle	0.85 (0.65–1.11)	1.42 (0.85–2.37)	*2.24 (1.22–4.12)	0.87 (0.62–1.23)
	Fourth	0.85 (0.64–1.13)	*1.97 (1.12–3.45)	1.88 (0.97–3.63)	0.83 (0.58–1.20)
	Richest	0.80 (0.56–1.14)	1.11 (0.62–2.00)	1.45 (0.63–3.34)	*0.56 (0.34–0.91)
Breastfeeding	No	Ref.	Ref.		
	Yes	1.23 (1.03–1.42)	0.90 (0.63–1.29)		
Wave×wealth index	Wave×second		*0.78 (0.64–0.96)		
	Wave×middle		*0.81 (0.66–1.00)		
	Wave×fourth		*0.73 (0.58–0.91)		
	Wave×richest		0.82 (0.66–1.03)		

† Wave 2, year 2000; wave 3, year 2006; wave 4, year 2011

* *p*<0.05

** *p*<0.01

*** *p*<0.001.

However, Kinh children had higher odds of experiencing illness with a cough than non-Kinh children (OR =1.29, CI=1.10–1.51). There was negative interaction between wealth and survey year (wave), suggesting that the impact of wealth status on illness with a cough decreased in each subsequent wave.

Mothers’ education level was significantly associated with seeking ORT. Children whose mothers attained lower secondary education and above had more than double the odds of receiving ORT during an episode of diarrhea, compared with children with mothers who attained primary or lower education. Children who were male (OR=1.61, CI=1.09–2.38) or who were from households in the middle wealth quintile (OR=2.24, CI=1.22–4.12) were more likely to receive ORT than children who were female or from households in the poorest wealth quintile. There were no significant interactions between any of the independent variables and survey year (wave) suggesting that the effects were similar in each wave. Seeking treatment through formal healthcare services was significantly associated only with household wealth. The odds ratio for the wealthiest compared with the poorest quintile was 0.56 (CI: 0.34–0.91).

## Discussion

This study on the determinants of diarrhea and illness with a cough and their respective treatments among young children in recent years in Vietnam has a few key findings.

First, we found an undesirable trend in seeking formal healthcare services for treating illness with a cough. Not only was the use of formal healthcare for illness with a cough low, but this decreased over time. Children's cough symptoms can be easily overlooked unless production of thick sputum or persistent over a long time (20, 21). Such misconceptions can put children at risk because mothers often think that formal treatment is not necessary. Nevertheless, if there are reliable, available, and affordable primary healthcare facilities, mothers may seek treatment. Yet, many Vietnamese increasingly mistrust public primary healthcare facilities mainly because the quality of public services in the community has fallen since the legalization of private medical practice in 1989. On the other hand, private healthcare is expensive. This may explain Vietnamese mothers’ reluctance to seek healthcare for illnesses that they do not perceive as being serious (21).

A second finding is that being of Kinh ethnicity was negatively associated with having recent diarrhea, but positively associated with illnesses with a cough. This may be explained by environmental factors. The occurrence of diarrhea is heavily dependent on hygiene and environmental factors such as unsafe water, polluted soil, and bad hygiene (22). Ethnic minorities in Vietnam, which account for only about 15% of the population, mainly reside in remote and mountainous areas where hygiene-related infrastructure and environmental conditions are often poor (23). Although environmental factors were already included in part through the wealth index, which measured household amenities related to sanitation, unclean environments outside the household can also promote infection which leads to diarrhea in children. The positive association between Kinh ethnicity and illness with a cough may be explained by the fact that people of Kinh ethnicity are more likely to live in more developed urban areas in which there is exposure to air pollution, which can lead to respiratory problems in children (24).

Household wealth effects over time are also noteworthy. Specifically, the protective effect of wealth for illness with a cough was higher in successive waves, consistent with previous studies that have demonstrated a negative relationship between poverty and respiratory diseases. Crowded living environments, non-immunization, and ignorance of preventive measures, all of which are common among poorer people, may have also been influential (25). As the gap between the rich and the poor widened in Vietnam during the study period, higher economic status may have been protective for illness with cough.

Another finding is that mothers’ education levels were significantly associated with seeking ORT, but not with seeking formal treatment for an illness with a cough. A plausible reason for this can be found in research conducted by Ali et al. in Bangladesh, who claimed that a main reason for not using ORT was because of the misconception that ORT can stop the diarrhea (9). The mothers with incorrect information about the benefits of ORT were half as likely to use ORT compared with mothers who understood correctly that ORG is not a cure for diarrhea but helps replace lost salt and water. On the other hand, one of the reasons why mothers do not seek formal healthcare services for illness with a cough is because of distrust of public services and non-affordability of private services.

An unexpected result was the significant negative association between household wealth and seeking formal healthcare for illness with a cough. Children from households in the richest wealth quintile were less likely to seek formal healthcare for illness with cough, compared to children in the poorest quintile, but they were more likely to use ORT for diarrhea. There are some studies that support a negative association between wealth and formal healthcare-seeking for illness with cough. For example, Taffa and Chepngeno (21) revealed that household income was significantly associated with healthcare-seeking behaviors only up to a certain threshold. For those children above the threshold, other factors, such as proper understanding of the disease, were important determinants. There are some possible underlying mechanisms that may be operating here. Those who are richer are more likely to be more highly educated (26–28). In Vietnam, many people choose self-treatment during the initial stage unless the symptoms were perceived as being too severe (10). For example, 40–60% of individuals initially depend on self-treatment using western or traditional medicines, and about 27.3% stock various medicines, including antibiotics, for self-medication (29). Wealthier mothers who are better educated may try to control cough symptoms by using high-quality self-medication at home. It is also possible that cough symptoms in children from wealthier households may be less serious during early stages of the disease process because of good nutritional environments. On the other hand, commercial ORS can be up to seven times more costly than homemade ORS (30). Given that children may have diarrhea for as many as 60 days a year, and diarrhea tends to spread easily among siblings in the same family, commercial ORS can be very costly for poorer families. This is therefore one reason why we might expect that children from rich households would be more likely to be treated with ORT.

Although this is the first study of its kind, there are some limitations. First, as mentioned before, ORT is effective only on watery diarrhea because it does not stop diarrhea. ORT prevents dehydration during a diarrhea episode. Therefore, we suggest that future questionnaires should include questions about the type or duration of diarrhea to more accurately measure proper treatment. Second, childhood diarrhea and illness with a cough are likely to be associated with community-level factors such as accessibility to clean water or air, sanitation facilities, and hygiene practices (31). Future research exploring the effects of contextual factors using multi-level modeling could help to further explain the associations described here. Finally, there are possibly many other determinants that are not available in this dataset.

## Conclusions

The findings of this study indicate the importance of raising awareness of childhood illnesses, such as diarrhea and illness with a cough, in Vietnam, and developing interventions that target at-risk groups, such as those who are from ethnic minorities, who have less wealth, and whose mothers have limited schooling. However, it is important that campaigns convey accurate messages and do not mislead. For example, aggressive marketing campaigns on ORT can wrongly convince people that ORT is effective on all kinds or diarrhea, and they promote unnecessary use of expensive commercial treatments. Qualitative research may also help to improve understanding of these and other issues. Future policies aimed at tackling childhood morbidity should include comprehensive strategies that impact on socioeconomic and environmental factors.

## Authors' contributions

All listed authors have contributed significantly to the manuscript and approved the final version for publication. HYL designed the concept, analyzed and interpreted data, and drafted the manuscript. NVH drafted the manuscript, gave critical comments, and edited the manuscript. SGC gave critical comments, revised the manuscript, and completed editing.

## Conflict of interest and funding

The authors declare there are no conflicts of interest.
